# Longitudinal changes in macular retinal layer thickness in pediatric populations: Myopic vs non-myopic eyes

**DOI:** 10.1371/journal.pone.0180462

**Published:** 2017-06-29

**Authors:** Scott A. Read, David Alonso-Caneiro, Stephen J. Vincent

**Affiliations:** Contact Lens and Visual Optics Laboratory, School of Optometry and Vision Science, Queensland University of Technology, Brisbane, Queensland, Australia; Charite Universitatsmedizin Berlin, GERMANY

## Abstract

Knowledge of the normal *in vivo* thickness of the retina, and its individual layers in pediatric populations is important for diagnosing and monitoring retinal disorders, and for understanding the eye’s normal development and the impact of eye growth and refractive error such as myopia (short-sightedness) upon retinal morphology. In this prospective, observational longitudinal study, total retinal thickness (and individual retinal layer thickness) and the changes in retinal morphology occurring over an 18-month period were examined in 101 children with a range of refractive errors. In childhood, the presence of myopia was associated with subtle but statistically significant (p<0.05) changes in the topographical thickness distribution of macular retinal thickness (and retinal layer thickness), characterised by a thinning of the parafoveal retina (and parafoveal or perifoveal thinning in most outer and inner retinal layers). The parafoveal retina was on average 6 μm thinner in myopic children. However, over 18 months, longitudinal changes in retinal thickness and individual layers were of small magnitude (average changes of less than 2 μm over 18 months), indicative of a high degree of stability in retinal morphology in healthy adolescent eyes, despite significant eye growth over this same period of time. This provides the first detailed longitudinal assessment of macula retinal layer morphology in adolescence, and delivers new normative data on expected changes in retinal structure over time and associated with myopia during this period of childhood development.

## Introduction

Myopia is a refractive error that occurs due to excessive axial elongation of the eye, and is one of the most common eye conditions affecting pediatric populations globally [1]. Due to its rising prevalence in recent decades and association with a range of ocular pathologies, myopia is considered to be a significant public health concern [2]. The increased risk of myopic eyes developing retinal complications later in life (e.g. retinal detachment, myopic maculopathy) [2] provides significant impetus to expand our understanding of the retinal changes associated with childhood myopia.

The ability of spectral domain optical coherence tomography (SD-OCT) to rapidly and non-invasively acquire high-resolution cross-sectional retinal images, has seen this imaging modality establish itself as an invaluable tool in evaluating the pediatric retina [3]. *In-vivo* imaging of the pediatric retina with OCT provides detailed morphological data, valuable in the diagnosis, monitoring and grading of a variety of retinal conditions [4–7], and in the assessment and monitoring of the effectiveness of treatments [8–10]. The diagnosis and monitoring of abnormalities with OCT typically involves a comparison between a patient’s global or local retinal morphology (and their changes over time) against a normal reference morphology (normative database). Therefore, the reliable clinical application and interpretation of OCT measures in pediatric populations relies upon a thorough knowledge of the normative range of structural characteristics of the retina (and retinal layers) in childhood, and the typical changes in structure expected to occur over time during normal childhood development.

Recent research utilising SD-OCT has substantially advanced our understanding of the normal developmental changes occurring in the *in vivo* structure of the total retina [11–14] and the retinal layers [15–21] throughout infancy [15–17,20] and childhood [11–14,18,19,21]. These studies have provided detailed information on the normal range of thickness in the retina and its layers and have demonstrated that substantial changes in retinal morphology (including a reorganisation and redistribution of the inner and outer retinal layers) occurs in normal children from birth and through infancy [15–17,20], with more subtle increases in total retinal thickness and layer thickness change also noted throughout childhood [12,18,21]. It should be noted though, that the vast majority of these studies of normal children have involved cross-sectional study designs, with only two studies, primarily focussed on measures of retinal thickness in neonates and infants [15,20], including some longitudinal measures of retinal thickness in a portion of their participants.

Since myopia typically manifests and progresses in childhood and adolescence, a number of recent studies have also used SD-OCT to examine the impact of childhood myopia and refractive error upon retinal thickness [12–14,19], providing insights into the potential role of the retina in the development of refractive error and the possible impact of the axial eye elongation associated with myopia development and progression upon retinal morphology. These studies have shown some subtle differences in macular retinal morphology in childhood associated with myopia, with a small magnitude (most studies report an average difference in thickness between myopic and emmetropic children of <10 μm) thinning of the retina in parafoveal and/or perifoveal retinal regions being a typical finding [14,19]. One recent study using long wavelength swept source OCT also examined the impact of myopia upon macular ganglion cell layer (GCL) and retinal nerve fibre layer (NFL) thickness and found myopic children exhibited a significantly thinner perifoveal GCL, but no significant difference in the NFL thickness associated with myopia [19]. To date, these previous studies examining retinal thickness (and retinal layer thickness) and its association with myopia in childhood have also involved only cross-sectional study designs [12–14,19], which limits the insights that can be drawn from the data regarding the time course of retinal changes associated with the development and progression of childhood myopia.

In this study, we aimed to expand knowledge of the changes in macular retinal layer thickness (and their topographical variations) associated with myopia in childhood, and the normal changes occurring in retinal layer thickness over time in a healthy pediatric population. We conducted a prospective longitudinal investigation of retinal layer thickness using SD-OCT in myopic and non-myopic children with normal vision.

## Materials and methods

This prospective, longitudinal study examining macular retinal layer thickness in childhood, involved the 102 children aged between 10–15 years, enrolled in the Role of Outdoor Activity in Myopia Study (ROAM Study). Retinal thickness measures (and a range of individual retinal layer thickness metrics) for each child were derived from SD-OCT images collected at 4 study visits, conducted every 6 months over an 18 month period. The study was approved by the Queensland University of Technology human research ethics committee (approval number: 1100001557) and all parents provided written informed consent, and children written assent prior to participation. All children enrolled in the study were treated in accordance with the tenets of the Declaration of Helsinki.

A detailed description of the study participants and procedures have been provided in a number of previous publications [22,23]. All children enrolled had normal vision in both eyes (logMAR visual acuity of 0.00 or better in each eye); no history or evidence of ocular disease, injury or surgery and no manifest hyperopic refractive errors of greater than +1.25 DS. One participant exhibited signs of developing macular neuroretinopathy at their second study visit and was therefore excluded from all analyses. The 101 subjects included in the final analysis had a mean age at the baseline visit of 13.1 ± 1.4 years, exhibited a mean spherical equivalent refraction of their right eye of -0.76 ± 1.67 D (range to -8.0 to +1.0 D) and mean cylindrical refraction of -0.21 ± 0.38 D (range -2.0 to 0.0 D). Fifty two percent of participants were female. Subjects were classified based upon their right eye’s non-cycloplegic spherical equivalent subjective refractive error (SER), as being either myopic (SER of -0.75 D or more myopia, mean SER -2.39 ± 1.51 D, n = 41) or non-myopic (SER less than +1.25 D and greater than -0.50 D, mean SER +0.35 ± 0.31 D, n = 60). Retention of subjects over the 18 month study was generally good, with 94 children (92% of enrolled participants) completing all 4 study visits. Three children were lost to follow-up (two after their baseline visit, and one after their second ocular measurement visit) and four children were excluded after they began orthokeratology contact lens wear (after their second (n = 3) or third (n = 1) ocular measurement visit).

### SD-OCT imaging

At each of the 4 study visits, a series of high resolution cross-sectional retinal images were collected from each child’s right eye using the Heidelberg Spectralis (Heidelberg Engineering, Heidelberg, Germany) SD-OCT instrument. This device uses a super luminescent diode of central wavelength of 870nm for OCT scanning, capturing 40,000 A-scans per second, in order to provide cross-sectional retinal OCT images with a digital axial resolution of 3.9 μm. Total retinal thickness [24] and individual retinal layer thickness [25] measures from this device are reported to be highly precise in normal adult subjects. OCT images were collected between 2pm and 5pm, to reduce the potential impact of ocular diurnal variations [26,27] upon the data.

**Fig 1:**
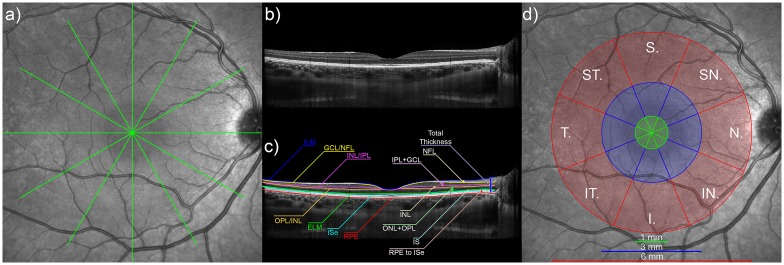
Overview of the OCT imaging and analysis procedures used in the study. At each study visit each child had 2 repeated series of OCT scans collected, each consisting of 6 radial scan lines centred on the fovea and separated by 30° (a). Each of the captured OCT images (example from the horizontal scan line is shown in [b]), were subsequently analysed using a semi-automated procedure to segment 8 retinal boundaries, including: the retinal pigment epithelium (RPE), inner segment ellipsoid (ISe), external limiting membrane (ELM), outer plexiform layer/inner nuclear layer (OPL/INL) boundary, inner nuclear layer/inner plexiform layer (INL/IPL) boundary, ganglion cell layer/nerve fibre layer (GCL/NFL) boundary and the inner limiting membrane (ILM) (c). These data from each scan were then used to derive 7 thickness measures including total retinal thickness, RPE to ISe, IS, ONL + OPL, INL, IPL + GCL, and NFL thickness. The average of each thickness metric was then calculated over the foveal (green zone in [d]), parafoveal (blue zone in [d]) and perifoveal (red zone in [d]), in superior (S), superior nasal (SN), nasal (N), inferior nasal (IN), inferior (I), inferior temporal (IT), temporal (T), superior temporal (ST) meridians (d).

Fig 1 provides an overview of the scanning protocol, and analysis procedures performed on the OCT images. At each study visit, 2 series of 6 high resolution radial OCT scan lines centred on the fovea and separated by 30° were captured using the instrument’s Enhanced Depth Imaging (EDI) mode (Fig 1a). Although EDI mode is typically used to enhance the visibility of the choroid, it has been shown that retinal thickness measures from EDI scans are comparable to those collected using the Spectralis instrument’s conventional imaging mode [28]. Frame averaging was also employed to improve the OCT image signal to noise ratio, using the instrument’s automatic real time eye tracking feature, and each OCT image was the average of 30 B-Scans. Only OCT images with a scan quality index of >20 dB were included (mean QI from all images analysed was 32.8 ± 2.6 dB). Four children (2 non-myopes and 2 myopes) were unable to maintain stable central fixation for the capture of all 6 radial OCT images, and therefore a single horizontal OCT scan centred on the fovea was collected at each visit for these subjects. The instrument’s auto-rescan feature was also used in order to register the scans from each subject at each visit to the same retinal location as their baseline scans.

Axial optical biometry (Lenstar LS 900, Haag Streit AG, Koeniz, Switzerland) measures were also collected at each of the 4 study visits, in order to assess axial length, central corneal thickness, anterior chamber depth and crystalline lens thickness. These measures were used to quantify each child’s axial eye growth over the 18 month study period, and to adjust the transverse scaling of the OCT scans at each visit in order to account for ocular magnification factors.

### OCT image analysis

Following image capture at each study visit, the exported OCT images were analysed using custom written software. Initially, an automated graph based method [21,29] was used to segment the boundaries of 8 different retinal layers, including: the outer boundary of the retinal pigment epithelium (RPE), the inner boundary of the inner segment ellipsoid zone (ISe), the inner boundary of the external limiting membrane (ELM), the boundary between the outer plexiform layer and inner nuclear layer (OPL/INL), the boundary between the inner nuclear layer and the inner plexiform layer (INL/IPL), the boundary between the ganglion cell layer and the nerve fibre layer (GCL/NFL) and the inner boundary of the inner limiting membrane (ILM) (Fig 1c). An experienced observer, masked to the demographic and refractive details of the participants, then checked the integrity of the automated segmentation of each boundary and manually corrected any segmentation errors. Each subject’s individual ocular biometry data from each study visit was then used to adjust the transverse scale of their OCT data to account for ocular magnification differences associated with children’s ocular dimensions using methods described previously [30]. This procedure ensured that thickness analyses both within- and between- subjects were derived from the same sized retinal regions.

The magnification corrected segmentation data from each of the OCT images were then used to derive the total retinal thickness (defined as the axial distance from the RPE to the ILM); and 3 thickness metrics describing the outer retinal layers: the RPE to ISe thickness (axial distance from the RPE to the ISe), the inner segment (IS) thickness (axial distance from the ISe to the ELM) and the ONL+OPL thickness (axial distance from the ELM to the OPL/INL); and 3 thickness metrics describing the inner retinal layers: INL thickness (axial distance from the OPL/INL to the INL/IPL), IPL+GCL thickness (the distance from the INL/IPL to the GCL/NFL) and NFL thickness (the distance from the GCL/NFL to the ILM) (Fig 1c). Data from each series of OCT scans collected at each study visit were then averaged in order to determine the mean thickness for each metric, in 8 different meridians (temporal, superior temporal, superior, superior nasal, nasal, inferior nasal, inferior and inferior temporal meridians) over a series of concentric annular zones (foveal, parafoveal and perifoveal zones) across the central 6 mm diameter surrounding foveal centre (Fig 1d). Since inner retinal layers are not present at foveal centre, only parafoveal and perifoveal thickness measures were derived for the inner retinal layer metrics.

### Statistical analysis

All statistical analyses were carried out using IBM SPSS Statistics Version 23. Initially, the repeatability of the imaging and measurement procedures for each of the retinal thickness metrics were assessed by comparing the thickness from each of the two repeated series of OCT scans collected at each study visit for each child, using the methods of Bland and Altman [31].

To examine the topographical variations in each of the thickness metrics in the myopic and non-myopic children, along with the longitudinal changes in thickness over the 18 months of the study, linear mixed model (LMM) analyses with restricted maximum likelihood estimation were used. The LMMs examined the effects of study visit time (in years from baseline visit), retinal zone (i.e. foveal, parafoveal or perifoveal zone) and meridian upon each of the considered retinal thickness metrics, assuming a first order autoregressive covariance structure. The effects of refractive error group, gender and subject age at the baseline visit were also examined in each of the LMMs. Individual subject’s slopes and intercepts were included as random effects in the model.

## Results

### Within-session measurement repeatability

**Fig 2:**
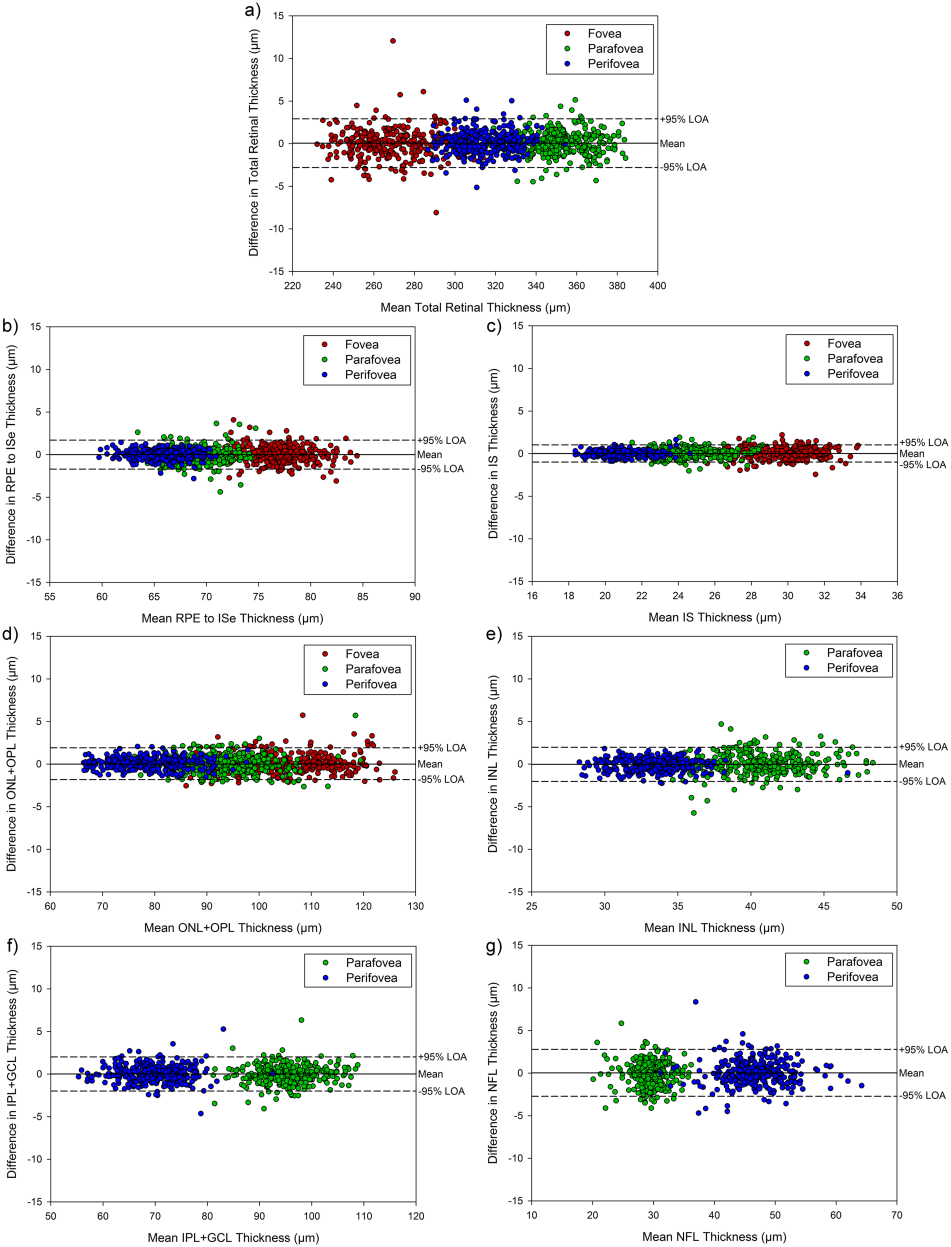
Bland-Altman plots demonstrating the repeatability of the total retinal thickness (a), RPE to ISe thickness (b), IS thickness (c), ONL+OPL thickness (d), INL thickness (e), IPL+GCL thickness (f), and NFL thickness (g) measures within each imaging session for each subject for each of the derived thickness metrics. The difference between the two repeated thickness estimates at each visit is plot against the mean of the two estimates. Solid line indicates the mean difference and dashed lines the 95% limits of agreement (LOA).

Fig 2 illustrates the Bland-Altman analysis examining the repeatability of each of the considered retinal thickness metrics from the two repeated scans collected at each imaging session for each subject in the study. The mean difference between repeated thickness measures was negligible for each of the different thickness metrics assessed, ranging from -0.02 μm (for INL thickness) to +0.06 μm (for total retinal thickness). Likewise, the 95% limits of agreement of these differences were also small for each of the thickness metrics, with the widest LOA being +2.9 to -2.8 μm for total retinal thickness and the narrowest LOA being +1.0 to -1.0 μm for the IS thickness. There also did not appear to be any systematic differences in measurement repeatability between the foveal, parafoveal and perifoveal zones, nor any relationship between the mean thickness value and the difference between repeated measures (Fig 2).

### Topographical thickness variations

#### Total retinal thickness

**Fig 3:**
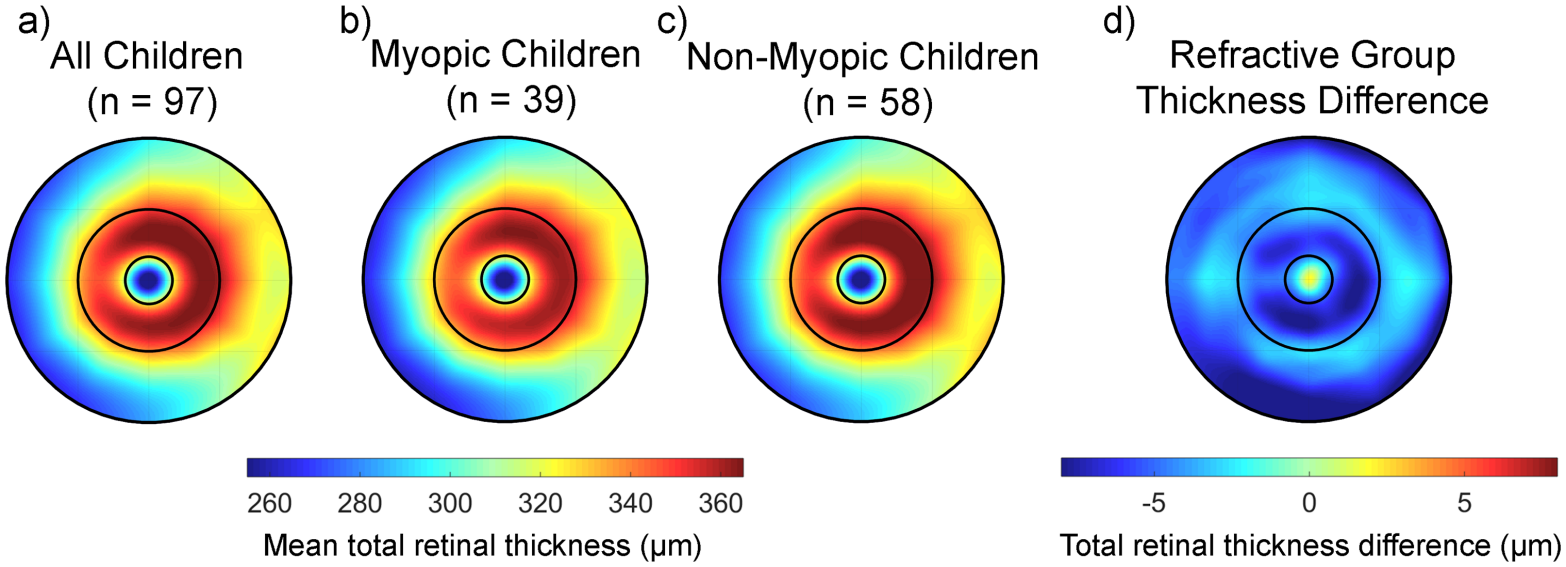
The average total retinal thickness at the baseline study visit across the central 6 mm in all children (a), the myopic children (b) and the non-myopic children (c). The difference in thickness between the myopic and non-myopic children is highlighted in (d). Negative values in (d) indicate a thinner retina in the myopic children and positive values indicate a thicker retina in the myopic children.

The topographical variations observed in total retinal thickness for all subjects at the baseline visit are illustrated in Fig 3 and Table 1. The retina was thinnest in the foveal zone (mean baseline thickness for all subjects was 268.0 ± 17.7 μm), and increased to a maximum in thickness in the parafoveal zone (mean 351.6 ± 14.4 μm), before reducing again in the perifoveal zone (313.2 ± 20.1 μm). In terms of meridians, the retina was found to be thickest in nasal meridians (318.8 ± 13.0 μm) compared to temporal (298.1 ± 11.9 μm), and thicker in superior (315.7 ± 11.1 μm) compared to inferior meridians (308.2 ± 12.0 μm). LMM analysis revealed the topographical variations in total retinal thickness were statistically significant, with variations in thickness observed with retinal zone (p < 0.001) and meridian (p < 0.001) (S1 Table reports the parameter estimates from the LMM analysis for the effects of retinal zone and meridian). Although there was no statistically significant difference in the overall mean retinal thickness of the myopic (mean thickness across all zones and meridians 308.8 ± 14.2 μm) and non-myopic children (mean thickness 312.4 ± 13.9 μm), there was a significant retinal zone by refractive group interaction (p < 0.001). Although on average myopic children exhibited a thinner retina than the non-myopic children at most locations, the largest differences in retinal thickness associated with refractive error were observed in the parafoveal (mean estimate of difference -5.9 μm [95%CI: -10.54 to -1.20 μm]) and perifoveal zones (mean estimate of difference -3.4 μm [95%CI: -8.13 to +1.31 μm]), compared to the foveal zone (mean estimate of difference -0.8 μm [95%CI: -5.52 to +3.92 μm]). However, it was only in the parafoveal zone that the differences associated with refractive error reached statistical significance (p < 0.02). Analyses including baseline axial length as a factor rather than refractive group also showed a significant axial length by retinal zone interaction (p < 0.001, parameter estimate for parafoveal zone -1.15 μm/mm [95% CI: -2.16 to -0.14 μm/mm]). On average, girls exhibited a slightly thinner total retina than boys (mean estimate of difference -4.6 μm [95%CI: -9.21 to -0.06 μm], p = 0.05). There were no significant effects of baseline age upon total retinal thickness (p > 0.05).

**Table 1:** Overview of the mean ± SD total retinal thickness at the study baseline visit in each of the eight meridians and three retinal zones assessed, for all subjects enrolled in the study.

		Mean ± SD Thickness (μm)			
		Foveal Zone	Parafoveal Zone	Perifoveal Zone	All Zones
Temporal	All Subjects	260.4 ± 19.1	337.4 ± 12.8	296.5 ± 13.0	298.1 ± 11.9
	Myopes	260.4 ± 18.7	335.0 ± 13.4	294.4 ± 14.5	296.6 ± 12.6
	Non-Myopes	260.3 ± 19.9	339.1 ± 12.3	298.0 ± 11.7	299.1 ± 11.5
Superior-Temporal	All Subjects	267.2 ± 17.1	348.8 ± 12.0	301.3 ± 12.7	305.8 ± 11.3
	Myopes	266.6 ± 17.6	345.2 ± 12.9	298.5 ± 13.8	303.4 ± 11.7
	Non-Myopes	267.6 ± 16.9	351.2 ± 10.9	303.1 ± 11.7	307.3 ± 10.8
Superior	All Subjects	270.1 ± 16.1	358.5 ± 12.7	318.5 ± 13.2	315.7 ± 11.1
	Myopes	270.2 ± 16.9	355.7 ± 13.0	316.4 ± 13.5	314.1 ± 11.3
	Non-Myopes	270.1 ± 15.7	360.5 ± 12.2	319.9 ± 12.9	316.8 ± 11.0
Superior-Nasal	All Subjects	270.3 ± 16.8	357.3 ± 12.7	332.5 ± 14.1	320.0 ± 11.8
	Myopes	270.9 ± 17.7	354.0 ± 12.1	329.9 ± 14.2	318.2 ± 11.6
	Non-Myopes	270.0 ± 16.3	359.5 ± 12.7	334.3 ± 13.9	321.3 ± 11.9
Nasal	All Subjects	268.9 ± 17.4	355.0 ± 13.6	332.6 ± 15.7	318.8 ± 13.0
	Myopes	268.4 ± 18.3	351.0 ± 12.4	330.7 ± 15.3	316.7 ± 12.1
	Non-Myopes	269.2 ± 16.9	357.7 ± 13.9	333.9 ± 16.0	320.2 ± 13.5
Inferior-Nasal	All Subjects	272.4 ± 17.5	355.2 ± 13.0	324.7 ± 14.6	317.5 ± 12.1
	Myopes	271.9 ± 18.4	351.3 ± 13.1	322.0 ± 15.3	315.1 ± 12.1
	Non-Myopes	272.8 ± 16.9	357.8 ± 12.4	326.6 ± 13.9	319.1 ± 11.9
Inferior	All Subjects	270.1 ± 17.3	352.6 ± 13.6	302.0 ± 13.8	308.2 ± 12.0
	Myopes	269.6 ± 18.9	348.7 ± 14.3	298.4 ± 15.2	305.6 ± 12.5
	Non-Myopes	270.5 ± 16.4	355.3 ± 12.4	304.4 ± 12.3	310.1 ± 11.4
Inferior-Temporal	All Subjects	265.1 ± 18.1	348.5 ± 13.0	297.5 ± 13.1	303.7 ± 11.8
	Myopes	264.4 ± 19.3	344.5 ± 13.6	294.1 ± 14.4	301.0 ± 12.1
	Non-Myopes	265.6 ± 17.3	351.2 ± 11.9	299.6 ± 11.4	305.5 ± 11.3
All Meridians	All Subjects	268.0 ± 17.7	351.6 ± 14.4	313.2 ± 20.1	311.0 ± 14.1
	Myopes	267.7 ± 18.6	348.1 ± 14.4	310.6 ± 20.7	308.8 ± 14.2
	Non-Myopes	268.2 ± 17.2	354.0 ± 13.9	315.0 ± 19.4	312.4 ± 13.9

#### Outer retinal layer thickness

**Fig 4:**
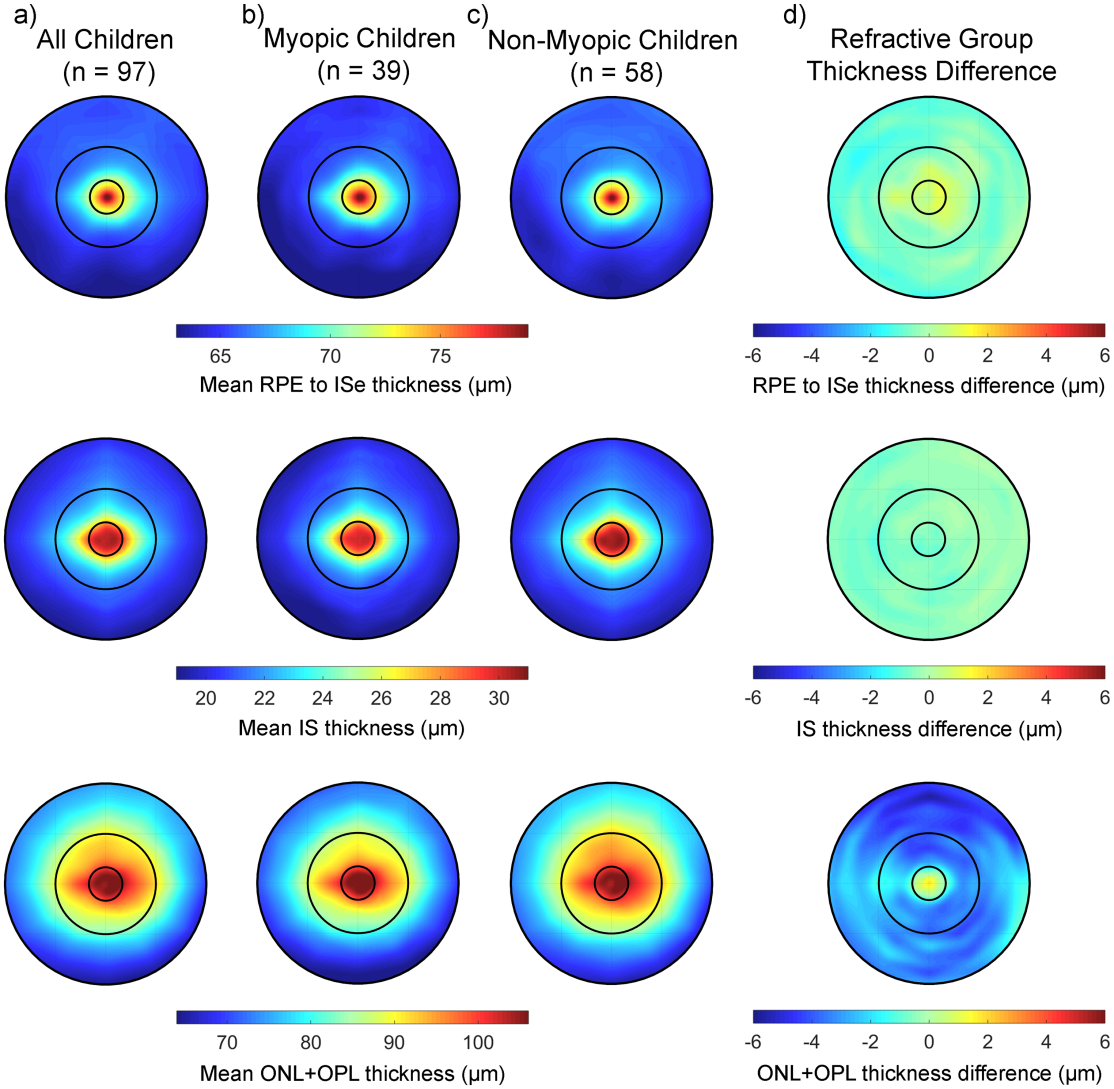
The average outer retinal thickness measures at the baseline study visit across the central 6 mm in all children (a), the myopic children (b) and the non-myopic children (c) for the RPE to ISe (top) IS (middle) and ONL+OPL (bottom) thickness metrics. The difference in thickness between the myopic and non-myopic children is highlighted in (d) for each metric. Negative values in (d) indicate a thinner retina in the myopic children and positive values indicate a thicker retina in the myopic children.

Statistically significant topographical variations in the thickness of each of the considered outer retinal layer metrics were also found (significant variations with both retinal zone and meridian, all p < 0.001) (Fig 4, Table 2). S2 Table reports the parameter estimates from the LMM analysis for the effects of retinal zone and meridian for the outer retinal layers. All of the outer retinal layers (RPE to ISe, IS and ONL+OPL thickness) exhibited their maximum thickness in the central foveal zone, and then reduced to a minimum in thickness in the perifoveal zone. As illustrated in Fig 4, meridional variations, although statistically significant for each of the outer retinal metrics were of small magnitude, indicative of a high degree of radial symmetry in the thickness variations. However, a consistent finding was that the inferior meridian was the thinnest for each of the considered outer retinal thickness metrics. There were no significant effects of baseline age on any of the outer retinal layer thickness metrics (all p > 0.05). IS thickness was the only outer retinal layer to exhibit a small magnitude difference in thickness associated with gender (estimated mean difference in thickness of -0.6 μm [95% CI: -1.0 μm to -0.1 μm] thinner in males) that reached statistical significance (p < 0.05).

**Table 2:** Overview of the mean ± SD retinal layer thickness at the study baseline visit in each of the eight meridians and three eccentricity zones assessed for all subjects enrolled in the study.

		Mean ± SD Thickness (μm)			
		Foveal Zone	Parafoveal ZoneZone	Perifoveal Zone	All Zones Zones
Temporal	RPE to ISe	77.1 ± 2.9	68.9 ± 2.7	64.7 ± 2.7	70.3 ± 2.4
	IS	30.5 ± 1.6	25.9 ± 1.6	21.2 ± 1.4	25.9 ± 1.3
	ONL+OPL	104.5 ± 9.2	97.9 ± 7.2	80.3 ± 6.8	94.2 ± 6.4
	INL		37.4 ± 3.7	34.1 ± 2.9	35.8 ± 2.8
	IPL+GCL		86.2 ± 6.1	74.1 ± 6.1	80.2 ± 5.1
	NFL		21.0 ± 2.4	22.0 ± 2.7	21.5 ± 2.2
Superior-Temporal	RPE to ISe	76.0 ± 2.4	67.6 ± 2.4	65.6 ± 2.4	69.8 ± 2.1
	IS	29.4 ± 1.6	23.6 ± 1.4	20.7 ± 1.2	24.6 ± 1.2
	ONL+OPL	104.5 ± 7.7	93.1 ± 6.8	80.4 ± 6.3	92.7 ± 5.9
	INL		39.6 ± 3.3	32.5 ± 2.5	36.0 ± 2.6
	IPL+GCL		96.5 ± 5.4	68.9 ± 5.3	82.7 ± 4.9
	NFL		28.3 ± 2.3	33.1 ± 3.8	30.7 ± 2.7
Superior	RPE to ISe	75.9 ± 2.7	67.9 ± 2.6	65.6 ± 2.2	69.8 ± 2.2
	IS	30.0 ± 1.6	24.3 ± 1.5	21.4 ± 1.2	25.2 ± 1.3
	ONL+OPL	104.0 ± 7.9	94.5 ± 7.1	82.1 ± 6.7	93.5 ± 6.0
	INL		41.0 ± 3.2	32.4 ± 2.5	36.7 ± 2.5
	IPL+GCL		97.4 ± 5.7	68.1 ± 4.9	82.7 ± 4.7
	NFL		33.5 ± 2.7	49.0 ± 6.1	41.2 ± 3.9
Superior-Nasal	RPE to ISe	76.9 ± 2.	68.8 ± 2.5	65.9 ± 2.3	70.5 ± 2.3
	IS	30.0 ± 1.7	24.1 ± 1.6	20.8 ± 1.1	25.0 ± 1.3
	ONL+OPL	104.8 ± 8.3	94.9 ± 6.8	81.5 ± 6.8	93.7 ± 6.0
	INL		40.6 ± 3.3	33.0 ± 2.6	36.8 ± 2.5
	IPL+GCL		97.3 ± 5.3	69.1 ± 5.8	83.2 ± 5.0
	NFL		31.5 ± 2.8	62.3 ± 6.0	46.9 ± 3.6
Nasal	RPE to ISe	77.6 ± 2.9	70.0 ± 2.4	65.9 ± 2.4	71.2 ± 2.2
	IS	30.5 ± 1.7	25.7 ± 1.8	21.1 ± 1.3	25.7 ± 1.3
	ONL+OPL	106.4 ± 8.5	98.6 ± 7.3	80.6 ± 7.2	95.2 ± 6.3
	INL		39.4 ± 3.1	35.3 ± 2.9	37.3 ± 2.3
	IPL+GCL		95.9 ± 6.5	79.3 ± 6.7	87.6 ± 5.5
	NFL		25.4 ± 2.4	50.4 ± 5.0	37.9 ± 2.9
Inferior-Nasal	RPE to ISe	76.9 ± 2.6	68.5 ± 2.3	64.6 ± 2.2	70.0 ± 2.1
	IS	30.1 ± 1.7	24.1 ± 1.5	20.3 ± 1.1	24.8 ± 1.3
	ONL+OPL	105.3 ± 7.9	93.3 ± 6.8	75.4 ± 6.4	91.3 ± 5.9
	INL		40.3 ± 2.8	32.2 ± 2.7	36.2 ± 2.2
	IPL+GCL		97.8 ± 5.4	67.2 ± 5.5	82.5 ± 4.6
	NFL		31.3 ± 2.7	64.9 ± 9.7	48.1 ± 5.4
Inferior	RPE to ISe	75.6 ± 2.6	66.9 ± 2.5	63.4 ± 2.3	68.6 ± 2.2
	IS	30.1 ± 1.6	23.9 ± 1.5	20.5 ± 1.2	24.8 ± 1.2
	ONL+OPL	103.9 ± 8.1	89.4 ± 7.0	72.2 ± 6.1	88.5 ± 5.9
	INL		42.8 ± 3.7	31.7 ± 2.8	37.2 ± 2.7
	IPL+GCL		96.3 ± 5.3	63.5 ± 5.0	79.9 ± 4.6
	NFL		33.3 ± 3.1	50.8 ± 6.4	42.1 ± 3.9
Inferior-Temporal	RPE to ISe	76.4 ± 2.6	67.6 ± 2.4	64.2 ± 2.4	69.4 ± 2.2
	IS	29.8 ± 1.6	23.8 ± 1.5	20.2 ± 1.1	24.6 ± 1.3
	ONL+OPL	102.2 ± 8.2	90.9 ± 6.5	75.6 ± 6.0	89.6 ± 5.8
	INL		40.3 ± 3.3	32.8 ± 2.5	36.5 ± 2.5
	IPL+GCL		97.2 ± 5.2	69.3 ± 5.5	83.2 ± 4.7
	NFL		28.7 ± 2.4	35.4 ± 3.7	32.0 ± 2.6
All Meridians	RPE to ISe	76.6 ± 2.7	68.3 ± 2.3	65.0 ± 2.5	70.0 ± 2.3
	IS	30.0 ± 1.5	24.4 ± 1.8	20.8 ± 1.3	25.1 ± 1.4
	ONL+OPL	104.5 ± 8.3	94.1 ± 7.5	78.6 ± 7.3	92.4 ± 6.4
	INL		40.2 ± 3.6	33.0 ± 2.9	36.6 ± 2.6
	IPL+GCL		95.5 ± 6.7	70.0 ± 7.2	82.8 ± 5.4
	NFL		29.1 ± 4.8	45.9 ± 15.0	37.5 ± 9.2

A number of small magnitude, but statistically significant differences in the thickness of each of the outer retinal layer metrics were also found to be associated with refractive group (Fig 4d). The RPE to ISe thickness exhibited significant refractive group by zone (p < 0.001) and refractive group by meridian (p < 0.01) interactions (but no overall differences in thickness associated with refractive group, p = 0.74). The myopic children exhibited a more rapid thinning in RPE to ISe thickness from the fovea to the perifovea (estimated mean change -12.3 μm [95% CI: -12.5 to -12.0 μm]) compared to the non-myopes (estimated mean change -11.2 μm [95% CI: -11.4 to -11.0 μm]). On average the IS thickness was marginally thinner in the myopes compared to the non-myopes (estimated mean difference -0.5 μm [95% CI: -0.92 to -0.02 μm]) and this difference reached statistical significance (p = 0.02), however there were no significant refractive group by zone or refractive group by meridian interactions. For the ONL+OPL thickness in the parafoveal and perifoveal zone, the myopic children exhibited significantly less thickness than the non-myopic children (estimated mean difference of -2.5 μm [95% CI: -4.9 to -0.1 μm] in the parafoveal zone and -2.8 μm [95% CI: -5.2 to -0.3 μm] in the perifoveal zone), with the largest magnitude differences found in the superior meridian of the perifoveal zone (estimated mean difference -3.6 μm [95% CI: -6.1 to -1.2 μm]) (refractive group by zone, refractive group by meridian interaction both p < 0.001).

#### Inner retinal layer thickness

**Fig 5:**
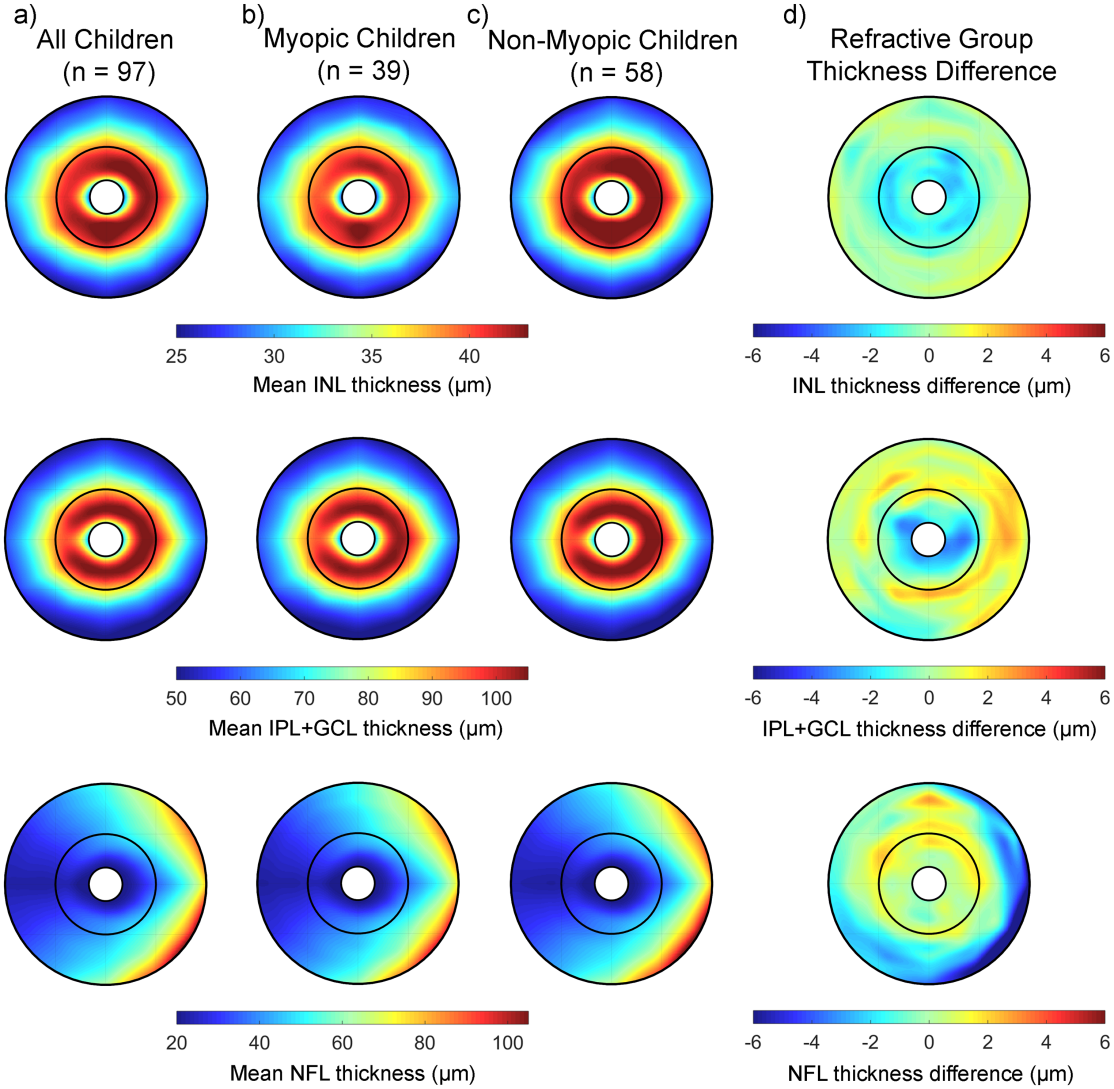
The average inner retinal thickness measures at the baseline study visit across the central 6 mm in all children (a), the myopic children (b) and the non-myopic children (c) for the INL (top) IPL+GCL (middle) and NFL (bottom) thickness metrics. The difference in thickness between the myopic and non-myopic children is highlighted in (d) for each thickness metric. Negative values in (d) indicate a thinner retina in the myopic children and positive values indicate a thicker retina in the myopic children. The central 1 mm foveal zone has been removed in all maps since, the inner retinal layers do not extend to foveal centre.

Topographical thickness variations were also observed in each of the inner retinal layers (Fig 5, Table 2). The parameter estimates from the LMM analysis for the effects of retinal zone and meridian are provided in S3 Table. Both the INL and IPL+GCL were found to be thicker in the parafovea (mean thickness of 40.2 ± 3.6 μm and 95.5 ± 6.7 μm for the INL and IPL+GCL respectively) compared to the perifovea (mean thickness of 33.0 ± 2.9 μm and 70.0 ± 7.2 μm for the INL and IPL+GCL), whereas the NFL exhibited its greatest thickness in the perifoveal region (mean thickness of 29.1 ± 4.8 μm and 45.9 ± 15.0 μm in the parafoveal and perifoveal zones respectively) (significant effect of retinal zone for all layers p < 0.001). Each of these retinal layers also tended to exhibit their lowest thickness in the temporal meridians, and greatest thickness in nasal meridians, with the NFL exhibiting peaks in thickness in the superior nasal and inferior nasal meridians (Fig 5). There were no significant effects of baseline age or gender upon the thickness for any of the inner retinal layers (all p > 0.05). Each of these layers also exhibited small magnitude, but statistically significant differences in thickness associated with refractive error (a significant refractive group by retinal zone interaction was observed for all inner retinal layer thickness metrics, all p < 0.05). For the INL, the myopic children exhibited thinner retinal layers in the parafoveal zone (estimated mean difference of -1.3 μm, for the INL [95% CI: -2.2 to -0.4 μm]), whereas the NFL was found to be thinner in the myopic children in the perifoveal zone (estimated mean difference of -2.0 μm [95% CI: -2.9 to -1.0 μm], and a maximum difference of -3.0 μm [95% CI: -4.0 to -1.9 μm] in the inferior nasal perifoveal meridian). The IPL+GCL thickness was significantly greater in the myopic children in the perifoveal zone only (estimated mean difference 2.1 μm [95% CI: +0.3 to +3.9 μm]).

### Longitudinal changes in retinal thickness

**Fig 6:**
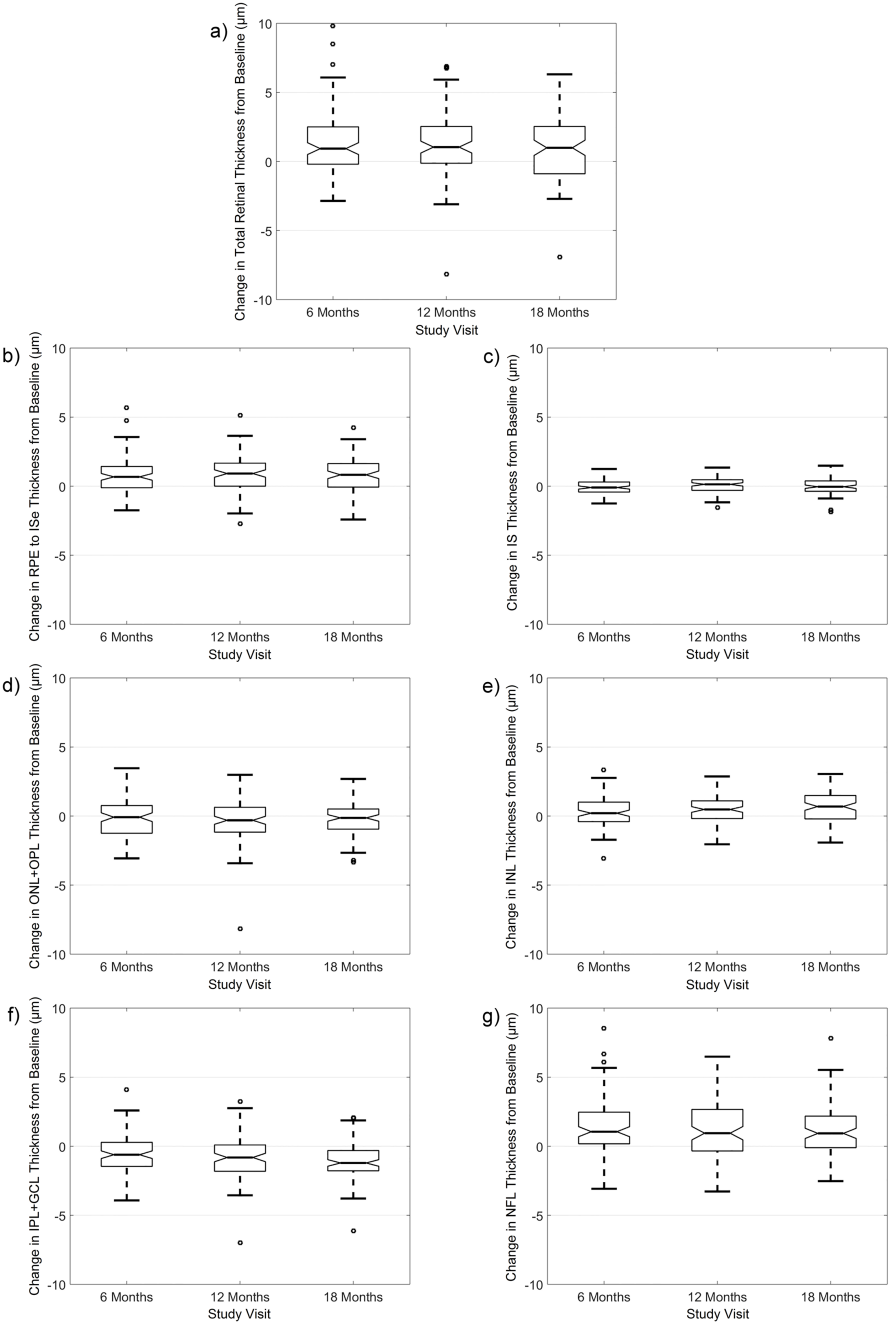
Notched box-plots illustrating the changes in total retinal thickness (a), RPE to ISe thickness (b), IS thickness (c), ONL+OPL thickness (d), INL thickness (e), IPL+GCL thickness (f), and NFL thickness (g) (averaged across all zones and meridians) from baseline at each visit over the 18 months study period. Solid horizontal line indicates the median, and box extends between the 25^th^ and 75^th^ percentile, whiskers extend to 1.5 times the interquartile range. The width of the notches in each box represents the 95% confidence interval of the median.

Table 3 presents the mean changes (from baseline) in each of the retinal thickness metrics after 18 months. The 5^th^ and 95^th^ percentiles of the observed changes in this population of healthy children are also presented to provide an overview of the range of thickness change observed over the study period. Over the 18 months of the study, only small magnitude changes in total retinal thickness were observed which were not statistically significant (mean change over 18 months across all considered zones was 0.8 ± 2.2 μm, p = 0.1). There was also no significant time by zone, time by meridian or time by refractive group (or axial length) interactions observed (all p > 0.05). The average changes observed in total retinal thickness at each of the study visits (compared to the baseline visit) are illustrated in Fig 6a.

**Table 3:** The mean ± SD changes from baseline over 18 months (and the normative reference range of these changes), in each of the retinal thickness metrics, across each of the considered macular zones for the normal children.

		Change in thickness from baseline over 18 months (μm)		
		Mean ± SD	Percentile	
			5^th^	95^th^
Total Retina	All Zones	+0.8 ± 2.2	-2.5	+4.4
	Foveal Zone	+0.7 ± 2.6	-3.4	+4.8
	Parafoveal Zone	+0.4 ± 2.7	-3.7	+4.2
	Perifoveal Zone	+1.4 ± 2.5	-2.5	+5.5
RPE to ISe	All Zones	+0.8 ± 1.2	-1.0	+2.8
	Foveal Zone	+0.9 ± 1.5	-1.6	+3.6
	Parafoveal Zone	+0.7 ± 1.4	-1.4	+3.0
	Perifoveal Zone	+0.9 ± 1.1	-1.1	+2.9
IS	All Zones	0.0 ± 0.6	-0.8	+1.1
	Foveal Zone	-0.1 ± 0.8	-1.3	1.2
	Parafoveal Zone	+0.1 ± 0.7	-0.9	1.4
	Perifoveal Zone	0.0 ± 0.5	-1.0	0.9
ONL+OPL	All Zones	+0.2 ± 1.2	-2.5	+1.7
	Foveal Zone	0.5 ± 1.7	-2.4	+3.5
	Parafoveal Zone	-1.2 ± 1.6	-4.0	+1.5
	Perifoveal Zone	0.0 ± 1.1	-2.1	+1.7
INL	All Zones	+0.6 ± 1.2	-1.1	+2.6
	Foveal Zone			
	Parafoveal Zone	+0.9 ± 1.6	-1.8	+3.5
	Perifoveal Zone	+0.4 ± 0.9	-1.0	+2.0
IPL+GCL	All Zones	-1.2 ± 1.4	-3.5	+1.4
	Foveal Zone			
	Parafoveal Zone	-0.8 ± 1.7	-4.0	+1.9
	Perifoveal Zone	-1.5 ± 1.6	-3.8	+1.4
NFL	All Zones	+1.2 ± 1.8	-1.0	+4.4
	Foveal Zone			
	Parafoveal Zone	+0.7 ± 1.8	-2.1	+4.5
	Perifoveal Zone	+1.6 ± 2.0	-1.0	+4.7

The magnitude of longitudinal change observed in each of the retinal layers was also small, however these small changes reached statistical significance for a number of the layers (Fig 6). Of the outer retinal layers (Fig 6b–6d), only RPE to ISe thickness was found to increase by a small but statistically significant amount over the 18 months of the study (mean change over 18 months was 0.8 ± 1.2 μm, p < 0.001), with slightly larger magnitude changes observed in the inferior (mean change of 1.4 ± 1.4 μm) and superior (mean change of 1.2 ± 1.6 μm) meridians (meridian by time interaction, p < 0.001). IS thickness and ONL+OPL thickness did not show any statistically significant changes over time (both p > 0.05). There were no significant interactions between thickness changes over time and refractive group or gender for any of the outer retinal layer thickness metrics (all p > 0.05).

The inner retinal layers were also seen to undergo small magnitude, statistically significant changes in thickness over the course of the study (visit time p < 0.001 for INL, IPL+GCL and NFL thickness) (Fig 6e–6g). INL thickness increased by a small amount over the 18 months (mean increase of 0.6 ± 1.2 μm), and these changes were most prominent in the parafoveal zone (mean increase of 0.9 ± 1.6 μm, zone by visit time interaction p = 0.002). Small increases in thickness were also observed in NFL thickness (mean increase of 1.2 ± 1.8 μm). Conversely, IPL+GCL thickness exhibited a small but statistically significant decrease over the course of the study (mean change of -1.2 ± 1.4 μm). No interactions between the thickness changes over time and gender were observed for any metrics (all p > 0.05).

**Fig 7:**
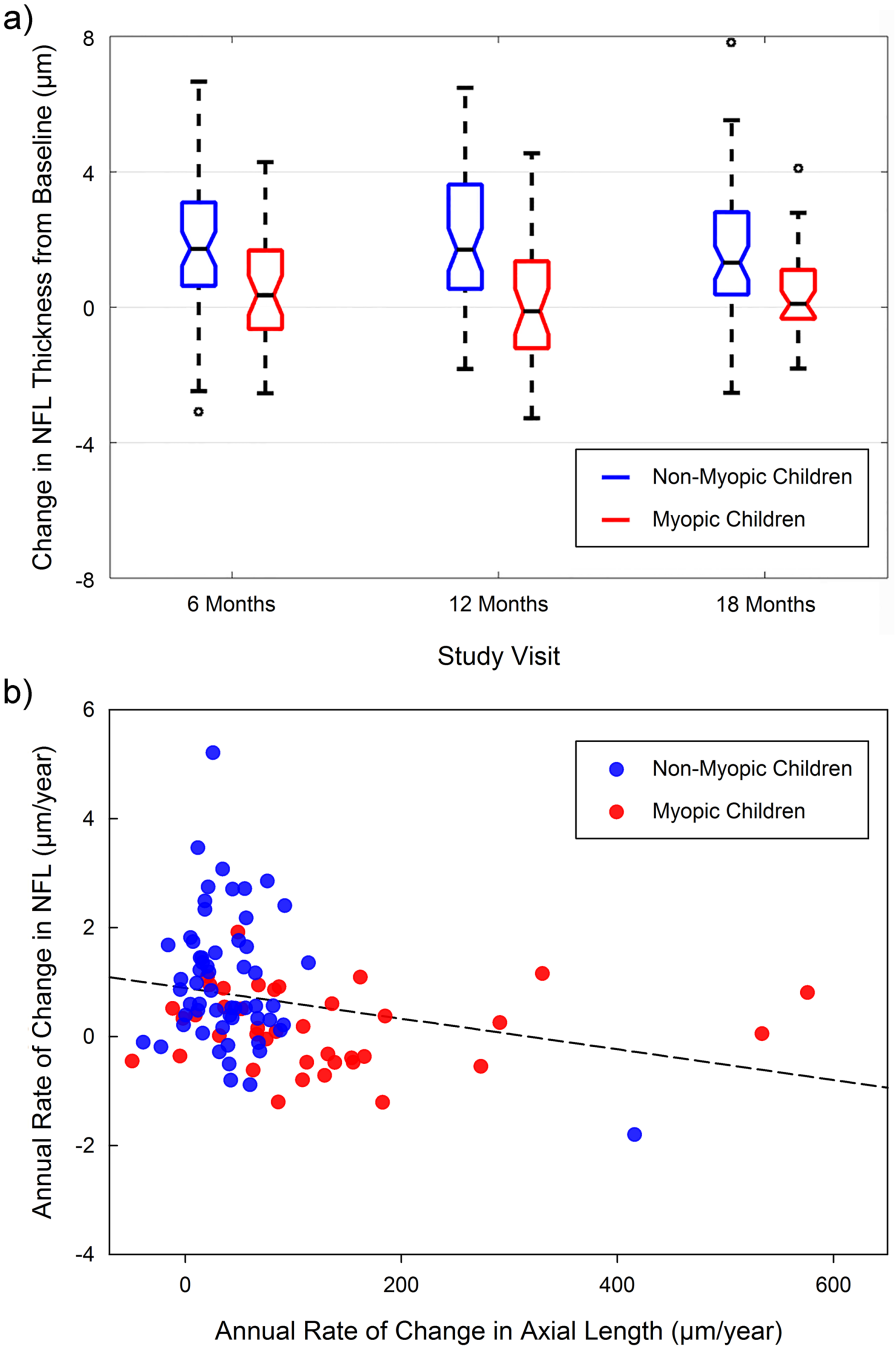
Notched box-plots illustrating the average change in NFL thickness (from baseline) at each visit in the study for the myopic (blue) and non-myopic (red) children in the study (solid horizontal line indicates the median change, and box extends between the 25^th^ and 75^th^ percentile, width of notches in each box represent the 95% confidence interval of the median, whiskers extend to 1.5 times the interquartile range of the data at each visit) (a) and the relationship between the rate of change in axial length and the rate of changes in NFL layer thickness over the course of the study (dashed line shows the best fit regression line) (b).

Of all of the considered thickness metrics, only NFL thickness exhibited a significant refractive group by visit time interaction (p = 0.001), with significantly greater increases in NFL thickness over time being observed in the non-myopic children (mean change of 1.6 ± 1.9 μm) compared to the myopic children (mean change of 0.4 ± 1.2 μm). Further analyses of these data revealed a significant association between the rate of axial eye growth, and the changes in NFL thickness, with greater axial eye growth being associated with less increase in NFL thickness over time (p < 0.05). Fig 7 illustrates the average changes in the NFL thickness in myopic and non-myopic children, and the relationship between the rate of axial eye growth and the NFL changes observed in the study.

We have previously documented significant longitudinal changes and variations associated with refractive group in the macular choroidal thickness of this population of children [20,28]. Correlation analysis revealed that there was no significant association between the longitudinal changes in choroidal thickness and the changes observed in any of the macular retinal layer metrics (all p > 0.05). Baseline choroidal thickness was also not significantly correlated with the considered retinal layer metrics (p>0.05), with the exception of INL thickness, which showed a weak but statistically significant positive association with choroidal thickness (r = 0.279, p < 0.01).

## Discussion

In this paper we have examined macular retinal thickness (and individual retinal layer thickness) using SD-OCT, in a population of 10–15 year old children with healthy eyes, normal vision and a range of refractive errors, and have explored the topographical thickness variations and the changes in thickness associated with myopia. Furthermore, we have also monitored the changes in retinal thickness and individual layer thickness longitudinally over an 18-month period of time, where this population of normal children were also documented to have undergone significant eye growth and changes in choroidal thickness [22,23]. The findings from this study further our understanding of the normal characteristics of macular retinal layer morphology in childhood, the differences in morphology associated with refractive error and the normal changes in retinal layer characteristics occurring over time in adolescence.

Although total retinal thickness did not show significant main effects of refractive group, the topographical thickness distribution of the total retina did show differences associated with the presence of myopia, with myopic subjects typically exhibiting a thinner retina, primarily in the parafoveal zone. These findings are indicative of a redistribution of retinal tissue thickness with myopia. We repeated each of the LMM analyses, using baseline axial length as a factor instead of refractive error group, and found similar trends of statistically significant associations between axial length and retinal thickness, indicating that the topographical changes in thickness associated with myopia are related to the increased axial length of myopic eyes. Although not a universal finding [12,13], previous studies using SD-OCT have also noted a thinning of the total retina in the parafoveal and/or the perifoveal region associated with myopia in childhood of similar magnitude to that found in the current study [14,19].

We also found small, but statistically significant differences in the thickness distribution of most of the outer and inner individual retinal layer metrics, with a thinner parafoveal or perifoveal zone found in the myopic children for most of the metrics examined. The outer retinal layer metrics, typically exhibited a small magnitude thinning in the perifoveal zones (with the exception of IS thickness which showed a small overall thinning in the myopic subjects), whereas inner retinal thickness metrics typically showed a small magnitude thinning in the parafoveal zone or perifoveal zone (with the exception of the IPL+GCL, that was thicker in myopes in the perifoveal zone). These findings suggest that the changes in total retinal thickness associated with myopia appear to be due to the combined effects of subtle changes in each of the retinal layers examined, rather than being driven by larger changes in a single layer. One previous study [19], has also examined the differences in macular retinal layer thickness associated with myopia in childhood, however they only examined the inner retinal layer thickness metrics of NFL and IPL+GCL thickness. Our current study, builds upon these previous findings by demonstrating small but statistically significant changes in layer thickness over a number of additional retinal layers. Jin et al [19] found that Chinese myopic children exhibited a thinner IPL+GCL thickness, in the perifoveal zone, and did not find a significant difference in NFL thickness associated with myopia in any considered zones. These differences from our current findings could potentially reflect differences associated with ethnicity, or may relate to the analytical methods used by Jin et al [19] who did not account for the effects of axial length upon transverse scan magnification, which could potentially mask some of the subtle regional differences in layer thickness with myopia observed in our current study.

It is noteworthy that although statistically significant, the average magnitude of thickness difference associated with myopia in our current study was generally small (the largest average magnitude of thinning of 6 μm was found for the total retina in the parafoveal zone) and unlikely to be of clinical significance. This suggests that in this population of young myopic subjects with mild to moderate levels of myopia (and significant differences in axial ocular biometrics to the non-myopic participants [22,23]), the changes in retinal morphology associated with refractive error are relatively subtle. In contrast to these small differences in retinal thickness associated with myopia, we have previously reported in this same population of children a substantial thinning of the macular choroid associated with myopia [22,30] (the subfoveal choroid was approximately 56 μm thinner in the myopic children). This suggests that choroidal changes associated with myopia appear to occur earlier and/or more rapidly in the process of childhood myopia development, compared to the more subtle retinal changes observed here.

Studies of adults have also documented reductions in total retinal thickness [32–34] and retinal layer thickness [33,34] in the parafoveal and perifoveal zones associated with myopia, typically of slightly higher magnitude than observed in our current study. The pattern of change in retinal layer thickness with myopia documented in adults appears to differ from what we have observed in children, with adult studies documenting evidence of thinning in inner (e.g. INL thickness) and outer retinal layers (e.g. ONL thickness) in the parafoveal and perifoveal zones, along with a small magnitude increase in other outer retinal layers (e.g. IS thickness) in this region associated with myopia. Although these studies have utilised different OCT instruments, scanning protocols and analytical approaches to our current study, these differences in layer thickness variations with myopia suggests that some redistribution and reorganisation of the retinal tissue layers continue during the progression of refractive error in later adolescence and early adulthood.

The total retinal thickness and each of the retinal layer thickness metrics examined showed significant topographical variations in thickness across the macular region. These thickness variations appear to be consistent with the previously documented topographical distribution and regional specialisation of retinal cells, and the pathways of the nerve fibres across the macula region [35–37]. The average values and topographical variations in total retinal thickness found in our current study are comparable with previous cross-sectional studies of pediatric populations using the same SD-OCT device [18]. Similarly the topographical distribution and average thickness values of the individual retinal layer thickness values in our current study also compare closely with previous studies assessing children of similar ages [18,19,21]. Studies of macular retinal layer thickness in healthy adults also report a similar thickness magnitude and pattern of topographical variation to our current study for the majority of layers examined, suggesting that the retinal morphology of the children in our current study was approaching adult dimensions [34,38,39].

We have also examined in detail, the longitudinal changes occurring in total retinal thickness, and individual layer thickness occurring in this population of normal children over an 18 month period. To date, only a limited number of studies have examined the longitudinal *in vivo* changes in retinal thickness occurring in childhood, and the majority of subjects examined in this previous work have been infants and very young children [15,20]. Our current study therefore provides new information regarding the normal thickness changes occurring over time in the retina and retinal layers of healthy eyes in adolescence. For total retinal thickness, no statistically significant changes were observed in our population of children over the study period, with a mean change of less than 1 μm observed over 18 months. This is in contrast to our previously documented significant increases in eye growth (a mean increase in axial length of 105 μm over 18 months for all 101 subjects [23]), and choroidal thickness (a mean increase of subfoveal choroidal thickness of 13 μm over 18 months was found for all subjects considered together) over time in this population of children [22]. Previous studies of total retinal thickness in childhood have noted substantial changes in total retinal thickness occurring throughout infancy and early childhood [20], with evidence that thickness changes appear to stabilise to adult levels by approximately the age of 12. This is consistent with our current longitudinal findings of no significant change in total retinal thickness over 18 months in adolescence.

Longitudinal analysis of the individual retinal layer thickness revealed a number of small magnitude, but statistically significant changes in retinal layer thickness over the 18 months of the study. Small magnitude increases in thickness were observed for the RPE to ISe, INL and NFL thickness metrics (up to a maximum mean increase of 1.6 μm for the NFL thickness in the perifoveal region over 18 months), and a small magnitude decrease in thickness was observed for the IPL+GCL thickness metrics (the largest mean decrease of 1.5 μm over 18 months was found in the perifoveal region). Although these statistically significant longitudinal changes in macular retinal layer thickness suggest the presence of some subtle re-organisation of the retinal tissue layers occurring in adolescence, the very small magnitude of these changes suggests a high degree of stability of the macular retinal layer thickness in the age of children examined in the study.

The retinal thickness values obtained for this population of healthy children over time provides novel normative data that can inform clinical practice and assist in the interpretation of clinical changes observed in pediatric macula OCT measures. The data presented in Table 3 provide an estimate of the normal range of changes expected over an 18 month period in macular retinal thickness metrics in healthy adolescents. These data suggest that (for the mean thickness change across each of the considered retinal zones) 95% of this population of children exhibited changes in total retinal thickness over 18 months between -4 μm and + 6 μm. Based upon these data, changes in total retinal thickness greater than this magnitude are unlikely to be found in the majority of normal children and should therefore raise clinical suspicion for the potential presence of retinal abnormalities. Examining these data for all of the considered individual retinal layer thickness metrics, shows the 5^th^ percentile of change in this population ranging from -1 to -4 μm and the 95^th^ percentile of change ranging from to +1 to +5 μm. Considering the largest range of changes across all of the considered layer metrics, a thinning of greater than 4 μm and a thickening of greater than 5 μm would be considered outside the normal range of change in retinal layers in this population of normal adolescent children. The small magnitude of these normative ranges emphasises the high measurement precision possible with SD-OCT, and the stability of the macular retinal layers in the population of children tested.

The longitudinal changes occurring in total retinal thickness and the majority of retinal layer thickness metrics examined were not significantly different between myopic and non-myopic children, despite the fact that the myopic children (mean increase in axial length of 184 μm over 18 months) exhibited significantly greater axial eye growth over time compared to the non-myopes (mean increase in axial length of 59 μm). This finding, coupled with the small magnitude of retinal change observed across all study visits, indicates that the magnitude of axial elongation observed in this population of children has limited effects upon the macular retinal morphology, and further suggests that retinal changes associated with myopia and axial elongation are relatively slow to manifest. Only one of the considered retinal layers (the NFL) showed a significant difference in longitudinal change between myopic and non-myopic children. While on average this layer showed a small but statistically significant increase in thickness over time, the myopic children showed significantly less increase in thickness compared to the non-myopic subjects. The changes in this layer were also related to the rate of axial eye growth, with greater axial elongation being associated with less NFL change over time. Although the small magnitude of these differences in NFL change means the clinical significance, and implications of this finding is unclear, it leaves open the possibility that longer term changes in NFL thickness associated with increased axial elongation could potentially play a role in the apparent increased susceptibility of adult myopic subjects to glaucomatous optic neuropathy [40].

Although our current study does provide the first detailed longitudinal analysis of macular retinal layer thickness in adolescence, a limitation of the study is the relatively short follow-up period of 18 months. Future studies with longer follow-up periods will expand our knowledge of the retinal changes expected in normal subjects throughout childhood and adolescence. The findings from our current study are also limited to the macular region of the retina, and while the macula is critically important for central vision, it does represent only a small proportion of the total retinal area. Future research utilising wide-field scanning methods is required to improve our knowledge of the normative morphology, and expected changes in morphology over time in childhood in more peripheral retinal regions. A further limitation of our data is that the retinal thickness metrics were derived from a 6-line radial scanning protocol, which was chosen to allow frame averaging to optimise scan quality while limiting the total scan time in this population of children. Although previous work examining macular layer thickness in children has also employed either a radial scanning protocol [19,21] or a single line scan [18] for analysis, it should be noted that more precise measures of topographical thickness distribution would be attained from a more densely sampled volumetric scanning protocol.

In summary, this longitudinal study has examined macular retinal thickness (and individual layer thickness) in a population of myopic and non-myopic children, and demonstrates subtle but statistically significant differences in the topographical thickness distribution associated with refractive error, characterised by a parafoveal retinal thinning (and parafoveal or perifoveal thinning in most outer and inner retinal layers) associated with myopia. Longitudinal retinal changes observed over the 18 month study were generally of small magnitude, indicative of a high degree of stability in total retinal thickness and retinal layer thickness in healthy adolescent eyes.

## Supporting information

S1 Dataset Macular retinal thickness data for each of the retinal layer metrics for all children enrolled in the study. (XLSX)

S1 Table Parameter estimates (and their 95% confidence intervals [CIs]) from the LMM analysis, for the fixed effects of retinal zone and retinal meridian upon total retinal thickness. (DOC)

S2 Table Parameter estimates (and their 95% confidence intervals [CIs]) from the LMM analysis, for the fixed effects of retinal zone and retinal meridian for the outer retinal layers. (DOC)

S3 Table Parameter estimates (and their 95% confidence intervals [CIs]) from the LMM analysis, for the fixed effects of retinal zone and retinal meridian for the inner retinal layers. (DOC)

## References

[1] Rudnicka AR, Kaptenakis VV, Wathern AK, Logan NS, Gilmartin B, Whincup PH, et al Global variations and time trends in the prevalence of childhood myopia, a systematic review and quantitative meta-analysis: implications for aetiology and early prevention. Br J Ophthalmol. 2016; 100: 882–890. doi: 10.1136/bjophthalmol-2015-307724 26802174 10.1136/bjophthalmol-2015-307724 PMC4941141

[2] Flitcroft DI. The complex interactions of retinal, optical and environmental factors in myopia aetiology. Prog Retin Eye Res. 2012; 31: 622–660. doi: 10.1016/j.preteyeres.2012.06.004 22772022 10.1016/j.preteyeres.2012.06.004

[3] Lee H, Proudlock FA, Gottlob I. Pediatric optical coherence tomography in clinical practice- Recent progress. Invest Ophthalmol Vis Sci. 2016; 57: OCT69–OCT79. doi: 10.1167/iovs.15-18825 27409508 10.1167/iovs.15-18825

[4] Gerth C, Zawadski RJ, Werner JS, Heon E. Retinal morphological changes of patients with x-linked retinoschisis evaluated by fourier-domain optical coherence tomography. Arch Ophthalmol. 2008; 126: 807–811. doi: 10.1001/archopht.126.6.807 18541843 10.1001/archopht.126.6.807 PMC2612690

[5] Maldonado RS, Toth CA. Optical coherence tomography in retinopathy of prematurity: Looking beyond the vessels. Clin Perinatol. 2013; 40: 271–296. doi: 10.1016/j.clp.2013.02.007 23719310 10.1016/j.clp.2013.02.007 PMC3947541

[6] Lee H, Purohit R, Sheth V, McLean RJ, Kohl S, Leroy BP, et al Retinal development in infants and young children with achromatopsia. Ophthalmology. 2015; 122: 2145–2147. doi: 10.1016/j.ophtha.2015.03.033 25972256 10.1016/j.ophtha.2015.03.033 PMC4582068

[7] McCafferty BK, Wilk MA, McAllister JT, Stepien KE, Dubis AM, Brilliant MH, et al Clinical insights into foveal morphology in albinism. J Pediatr Ophthalmol Strabismus. 2015; 52: 167–172. 10.3928/01913913-20150427-06 PMC4948980 26053207

[8] Cao C, Markovitz M, Ferenczy S, Shields CL. Hand-held spectral-domain optical coherence tomography of small macular retinoblastoma in infants before and after chemotherapy. J Pediatr Ophthalmol Strabismus. 2014; 51: 230–234. 25922867 10.3928/01913913-20140603-01

[9] Henry CR, Sisk RA, Tzu JH, Albini TA, Davis JL, Murray TG, et al Long-term follow-up of intravitreal bevacizumab for the treatment of pediatric retinal and choroidal diseases. J AAPOS. 2015; 19: 541–548. doi: 10.1016/j.jaapos.2015.09.006 26691034 10.1016/j.jaapos.2015.09.006

[10] Samara WA, Pointdujour-Lim R, Say EAT, Shields CL. Foveal microanatomy documented by SD-OCT following treatment of advanced retinoblastoma. J AAPOS. 2015; 19: 368–372. doi: 10.1016/j.jaapos.2015.02.019 26235789 10.1016/j.jaapos.2015.02.019

[11] Turk A, Ceylan OM, Arici C, Keskin S, Erdurman C, Durukan AH, et al Evaluation of the nerve fiber layer and macula in the eyes of healthy children using spectral domain optical coherence tomography. Am J Ophthalmol. 2012; 153: 552–559. doi: 10.1016/j.ajo.2011.08.026 22019223 10.1016/j.ajo.2011.08.026

[12] Barrio-Barrio J, Noval S, Galdós M, Ruiz-Canela M, Bonet E, Capote M, et al Multicenter Spanish study of spectral-domain optical coherence tomography in normal children. Acta Ophthalmol. 2013; 91: e56–e63. doi: 10.1111/j.1755-3768.2012.02562.x 23347665 10.1111/j.1755-3768.2012.02562.x

[13] Chen S, Wang B, Dong N, Ren X, Zhang T, Xiao L. Macular measurements using spectral-domain optical coherence tomography in Chinese myopic children. Invest Ophthalmol Vis Sci. 2014; 55: 7410–7416. doi: 10.1167/iovs.14-13894 25316719 10.1167/iovs.14-13894

[14] Li T, Zhou X, Wang Z, Zhu J, Shen W, Jiang B. Assessment of retinal and choroidal measurements in Chinese school-age children with Cirrus-HD optical coherence tomography. PLoS One. 2016; 11: e0158948 doi: 10.1371/journal.pone.0158948 27391015 10.1371/journal.pone.0158948 PMC4938617

[15] Maldonado RS, O'Connell RV, Sarin N, Freedman SF, Wallace DK, Cotten CM, et al Dynamics of human foveal development after premature birth. Ophthalmology. 2011; 118: 2315–2325. doi: 10.1016/j.ophtha.2011.05.028 21940051 10.1016/j.ophtha.2011.05.028 PMC3496560

[16] Dubis AM, Costakos DM, Subramaniam CD, Godara P, Wirostko WJ, Carroll J, et al Evaluation of normal human foveal development using optical coherence tomography and histologic examination. Arch Ophthalmol. 2012; 130: 1291–1300. doi: 10.1001/archophthalmol.2012.2270 23044942 10.1001/archophthalmol.2012.2270 PMC3724218

[17] Vajzovic L, Hendrickson AE, O'Connell RV, Clark LA, Tran-Viet D, Possin D, et al Maturation of the human fovea: Correlation of spectral domain optical coherence tomography findings with histology. Am J Ophthalmol. 2012; 154: 779–789. doi: 10.1016/j.ajo.2012.05.004 22898189 10.1016/j.ajo.2012.05.004 PMC3612897

[18] Yanni SE, Wang J, Cheng CS, Locke KI, Wen Y, Birch DG, et al Normative reference ranges for the retinal nerve fiber layer, macula, and retinal layer thicknesses in children. Am J Ophthalmol. 2013; 155: 354–360. doi: 10.1016/j.ajo.2012.08.010 23127751 10.1016/j.ajo.2012.08.010 PMC3545013

[19] Jin P, Zou H, Zhu J, Xu X, Jin J, Chang TC, et al Choroidal and retinal thickness in children with different refractive status measured by swept-source optical coherence tomography. Am J Ophthalmol. 2016; 168: 164–176. doi: 10.1016/j.ajo.2016.05.008 27189931 10.1016/j.ajo.2016.05.008

[20] Lee H, Purohit R, Patel A, Papageorgiou E, Sheth V, Maconachie G, et al In vivo foveal development using optical coherence tomography. Invest Ophthalmol Vis Sci. 2015; 56: 4537–4545. doi: 10.1167/iovs.15-16542 26200492 10.1167/iovs.15-16542

[21] Read SA, Collins MJ, Vincent SJ, Alonso-Caneiro D. Macular retinal layer thickness in childhood. Retina. 2015; 35: 1223–1233. doi: 10.1097/IAE.0000000000000464 25650708 10.1097/IAE.0000000000000464

[22] Read SA, Alonso-Caneiro D, Vincent SJ, Collins MJ. Longitudinal changes in choroidal thickness and eye growth in childhood. Invest Ophthalmol Vis Sci. 2015; 56: 3103–3112. doi: 10.1167/iovs.15-16446 26024094 10.1167/iovs.15-16446

[23] Read SA, Collins MJ, Vincent SJ. Light exposure and eye growth in childhood. Invest Ophthalmol Vis Sci. 2015; 56: 6779–6787. doi: 10.1167/iovs.14-15978 26567790 10.1167/iovs.14-15978

[24] Wolf-Schnurrbusch UE, Ceklic L, Brinkmann CK, Iliev ME, Frey M, Rothenbuehler SP, et al Macular thickness measurements in healthy eyes using six different optical coherence tomography instruments. Invest Ophthalmol Vis Sci. 2009; 50: 3432–3437. doi: 10.1167/iovs.08-2970 19234346 10.1167/iovs.08-2970

[25] Ctori I, Huntjens B. Repeatability of foveal measurements using Spectralis optical coherence tomography segmentation software. PLoS One. 2015; 10: e0129005 doi: 10.1371/journal.pone.0129005 26076457 10.1371/journal.pone.0129005 PMC4468112

[26] Read SA, Collins MJ, Alonso-Caneiro D. Diurnal variation of retinal thickness with spectral domain OCT. Optom Vis Sci. 2012; 89: 611–619. doi: 10.1097/OPX.0b013e3182501917 22446718 10.1097/OPX.0b013e3182501917

[27] Chakraborty R, Read SA, Collins MJ. Diurnal variations of axial length, choroidal thickness, intraocular pressure and ocular biometrics. Invest Ophthalmol Vis Sci. 2011; 52: 5121–5129. doi: 10.1167/iovs.11-7364 21571673 10.1167/iovs.11-7364

[28] Park SY, Kim SM, Song Y-M, Sung J, Ham D-I. Retinal thickness and volume measured with enhanced depth imaging optical coherence tomography. Am J Ophthalmol. 2013; 156: 557–566. doi: 10.1016/j.ajo.2013.04.027 23769194 10.1016/j.ajo.2013.04.027

[29] Chiu SJ, Li XT, Nicholas P, Toth CA, Izatt JA, Farsiu S. Automatic segmentation of seven retinal layers in SDOCT images congruent with expert manual segmentation. Optics Express. 2010; 18: 19413–19428. doi: 10.1364/OE.18.019413 20940837 10.1364/OE.18.019413 PMC3408910

[30] Read SA, Collins MJ, Vincent SJ, Alonso-Caneiro D. Choroidal thickness in myopic and nonmyopic children assessed with enhanced depth imaging optical coherence tomography. Invest Ophthalmol Vis Sci. 2013; 54, 7578–7586. doi: 10.1167/iovs.13-12772 24176903 10.1167/iovs.13-12772

[31] Bland JM, Altman DG. Measuring agreement in method comparison studies. Stat Methods Med Res. 1999; 8: 135–160. 10501650 10.1177/096228029900800204

[32] Harb E, Hyman L, Fazzari M, Gwiazda J, Marsh-Tootle W, COMET Study Group. Factors associated with macular thickness in the COMET myopic cohort. Optom Vis Sci. 2012; 89: 620–631. doi: 10.1097/OPX.0b013e318251293a 22525127 10.1097/OPX.0b013e318251293a PMC3348261

[33] Park S, Kim SH, Park TK, Ohn Y-H. Evaluation of structural and functional changes in non-pathologic myopic fundus using multifocal electroretinogram and optical coherence tomography. Doc Ophthalmol. 2013; 126: 199–210. doi: 10.1007/s10633-013-9375-0 23471724 10.1007/s10633-013-9375-0

[34] Liu X, Shen M, Yuan Y, Huang S, Zhu D, Ma Q, et al Macular thickness profiles of intraretinal layers in myopia evaluated by ultrahigh-resolution optical coherence tomography. Am J Ophthalmol. 2015; 160: 53–61. doi: 10.1016/j.ajo.2015.03.012 25800454 10.1016/j.ajo.2015.03.012

[35] Curcio CA, Allen KA. Topography of ganglion cells in human retina. J Comp Neurol. 1990; 300: 5–25. doi: 10.1002/cne.903000103 2229487 10.1002/cne.903000103

[36] Curcio CA, Sloan KR, Kalina RE, Hendrickson AE. Human photoreceptor topography. J Comp Neurol. 1990; 292: 497–523. doi: 10.1002/cne.902920402 2324310 10.1002/cne.902920402

[37] Fitzgibbon T, Taylor SF. Retinotopy of the human retinal nerve fibre layer and optic nerve head. J Comp Neurol. 1996; 375: 238–251. 8915828 10.1002/(SICI)1096-9861(19961111)375:2<238::AID-CNE5>3.0.CO;2-3

[38] Srinivasan VJ, Monson BK, Wojtkowski M, Bilonick RA, Gorczynska I, Chen R, et al Characterization of outer retinal morphology with high speed, ultrahigh-resolution optical coherence tomography. Invest Ophthalmol Vis Sci. 2008; 49: 1571–1579. doi: 10.1167/iovs.07-0838 18385077 10.1167/iovs.07-0838 PMC2846094

[39] Hood DC, Lin CE, Lazow MA, Locke KG, Zhang X, Birch DG. Thickness of receptor and post-receptor retinal layers in patients with retinitis pigmentosa measured with frequency domain optical coherence tomography. Invest Ophthalmol Vis Sci. 2009; 50: 2328–2336. doi: 10.1167/iovs.08-2936 19011017 10.1167/iovs.08-2936 PMC2835526

[40] Shen L Melles RB, Metlapally R, Barcellos L, Schaefer C, Risch N, et al The association of refractive error with glaucoma in a multiethnic population. Ophthalmology. 2016; 123: 92–101. doi: 10.1016/j.ophtha.2015.07.002 26260281 10.1016/j.ophtha.2015.07.002 PMC4695304

