# Supplementary material for: Longitudinal changes in macular retinal layer thickness in pediatric populations: Myopic vs non-myopic eyes
Source: PLoS One. 2017 Jun 29;12(6):e0180462. doi: 10.1371/journal.pone.0180462 (PMC5491256; doi:10.1371/journal.pone.0180462)
Supplement: S1 Table — (DOC) [file pone.0180462.s002.doc]

**S1 Table. Parameter estimates (and their 95% confidence intervals [CIs]) from the LMM analysis, for the fixed effects of retinal zone and retinal meridian upon** total retinal thickness

| Parameter | Estimate | Significance  (p-value) | 95% CI | |
| --- | --- | --- | --- | --- |
|  |  |  | Lower | Upper |
| Intercept | 298.92 | <0.001 | 294.03 | 302.69 |
| Retinal Zone  Foveal  Parafoveal  Perifoveal | -33.85  45.33  0* | <0.001  <0.001  - | -35.49  43.82  - | -31.18  47.46  - |
| Retinal Meridian  Superior  Superior Nasal  Nasal  Inferior Nasal  Inferior  Inferior Temporal  Temporal  Superior Temporal | 16.45  30.28  29.59  22.25  0.20  -4.08  -4.32  0* | <0.001  <0.001  <0.001  <0.001  0.817  <0.001  <0.001  - | 14.70  28.47  27.79  20.47  -1.48  -5.57  -5.45  - | 18.20  32.09  31.40  24.02  1.87  -2.60  -3.20  - |

* Parameter estimate set to zero since it is the reference level for this fixed effect.
