# Supplementary material for: Longitudinal changes in macular retinal layer thickness in pediatric populations: Myopic vs non-myopic eyes
Source: PLoS One. 2017 Jun 29;12(6):e0180462. doi: 10.1371/journal.pone.0180462 (PMC5491256; doi:10.1371/journal.pone.0180462)
Supplement: S2 Table — (DOC) [file pone.0180462.s003.doc]

**S2 Table. Parameter estimates (and their 95% confidence intervals [CIs]) from the LMM analysis, for the fixed effects of retinal zone and retinal meridian for the outer retinal layers.**

| Thickness  Metric | Parameter | Estimate | Significance  (p-value) | 95% CI | |
| --- | --- | --- | --- | --- | --- |
|  |  |  |  | Lower | Upper |
| RPE to IsE | Intercept | 64.95 | <0.001 | 64.14 | 65.76 |
|  | Retinal Zone  Foveal  Parafoveal  Perifoveal | 11.12  2.63  0* | <0.001  <0.001  - | 10.76  2.29  - | 11.48  2.96  - |
|  | Retinal Meridian  Superior  Superior Nasal  Nasal  Inferior Nasal  Inferior  Inferior Temporal  Temporal  Superior Temporal | 0.20  0.51  0.31  -0.63  -1.91  -1.31  -0.68  0* | 0.234  0.004  0.086  0.001  <0.001  <0.001  <0.001  - | -0.13  0.16  -0.04  -0.99  -2.63  -1.63  -0.94  - | 0.53  0.87  0.67  -0.27  -1.56  -0.98  -0.41  - |
| IS | Intercept | 20.68 | <0.001 | 20.22 | 21.13 |
|  | Retinal Zone  Foveal  Parafoveal  Perifoveal | 8.66  2.86  0* | <0.001  <0.001  - | 8.45  2.66  - | 8.87  3.06  - |
|  | Retinal Meridian  Superior  Superior Nasal  Nasal  Inferior Nasal  Inferior  Inferior Temporal  Temporal  Superior Temporal | 0.65  0.18  0.41  -0.47  -0.41  -0.56  0.44  0* | <0.001  0.098  <0.001  <0.001  <0.001  <0.001  <0.001  - | 0.45  -0.03  0.19  -0.68  -0.62  -0.75  0.29  - | 0.84  0.39  0.62  -0.26  -0.20  -0.36  0.60  - |
| ONL+OPL | Intercept | 77.69 | <0.001 | 75.32 | 80.05 |
|  | Retinal Zone  Foveal  Parafoveal  Perifoveal | 27.23  13.29  0* | <0.001  <0.001  - | 26.11  12.38  - | 28.35  14.20  - |
|  | Retinal Meridian  Superior  Superior Nasal  Nasal  Inferior Nasal  Inferior  Inferior Temporal  Temporal  Superior Temporal | 1.37  1.23  0.46  -4.75  -8.17  -4.48  0.05  0* | 0.001  0.004  0.287  <0.001  <0.001  <0.001  0.848  - | 0.56  0.40  -0.38  -5.58  -8.95  -5.18  -0.48  - | 2.17  2.07  1.29  -3.93  -7.38  -3.79  0.58  - |

* Parameter estimate set to zero since it is the reference level for this fixed effect.
