# Supplementary material for: Longitudinal changes in macular retinal layer thickness in pediatric populations: Myopic vs non-myopic eyes
Source: PLoS One. 2017 Jun 29;12(6):e0180462. doi: 10.1371/journal.pone.0180462 (PMC5491256; doi:10.1371/journal.pone.0180462)
Supplement: S3 Table — (DOC) [file pone.0180462.s004.doc]

**S3 Table. Parameter estimates (and their 95% confidence intervals [CIs]) from the LMM analysis, for the fixed effects of retinal zone and retinal meridian for the inner retinal layers.**

| Thickness  Metric | Parameter | Estimate | Significance  (p-value) | 95% CI | |
| --- | --- | --- | --- | --- | --- |
|  |  |  |  | Lower | Upper |
| INL | Intercept | 31.84 | <0.001 | 30.90 | 32.77 |
|  | Retinal Zone  Parafoveal  Perifoveal | 6.44  0* | <0.001  - | 5.98  - | 6.90  - |
|  | Retinal Meridian  Superior  Superior Nasal  Nasal  Inferior Nasal  Inferior  Inferior Temporal  Temporal  Superior Temporal | 0.05  0.47  2.83  -0.15  -0.59  0.35  1.91  0* | 0.179  0.110  <0.001  0.608  0.046  0.220  <0.001  - | -0.50  -0.11  2.25  -0.74  -1.17  -0.21  1.44  - | 0.60  1.05  3.42  0.44  -0.01  0.90  2.38  - |
| IPL+GCL | Intercept | 69.02 | <0.001 | 67.21 | 70.83 |
|  | Retinal Zone  Parafoveal  Perifoveal | 25.58  0* | <0.001  - | 24.80  - | 26.37  - |
|  | Retinal Meridian  Superior  Superior Nasal  Nasal  Inferior Nasal  Inferior  Inferior Temporal  Temporal  Superior Temporal | -0.79  0.46  9.79  -1.65  -5.56  0.17  5.27  0* | 0.092  0.351  <0.001  0.001  <0.001  0.712  <0.001  - | -1.72  -0.51  8.81  -2.64  -6.54  -0.75  4.50  - | 0.13  1.44  10.77  -0.65  -4.58  1.10  6.04  - |
| NFL | Intercept | 33.80 | <0.001 | 32.61 | 34.99 |
|  | Retinal Zone  Parafoveal  Perifoveal | -4.24  0* | <0.001  - | -5.09  - | -3.39  - |
|  | Retinal Meridian  Superior  Superior Nasal  Nasal  Inferior Nasal  Inferior  Inferior Temporal  Temporal  Superior Temporal | 16.09  28.16  16.65  30.63  17.07  1.69  -11.37  0* | <0.001  <0.001  <0..001  <0.001  <0.001  0.001  <0.001  - | 15.06  27.08  15.56  29.53  15.99  0.66  -12.24  - | 17.11  29.24  17.73  31.73  18.16  2.73  -10.50  - |

* Parameter estimate set to zero since it is the reference level for this fixed effect.
